# Immune Responses and Performance Are Influenced by Respiratory Vaccine Antigen Type and Stress in Beef Calves

**DOI:** 10.3390/ani10071119

**Published:** 2020-06-30

**Authors:** Rachel E. Hudson, Dexter J. Tomczak, Emily L. Kaufman, Ashlee M. Adams, Jeffery A. Carroll, Paul R. Broadway, Michael A. Ballou, John T. Richeson

**Affiliations:** 1 Department of Agricultural Sciences, West Texas A&M University, Canyon, TX 79016, USA; rachel.hudson@ttuhsc.edu (R.E.H.); dextert@frionaindustries.com (D.J.T.); emily.louann.kaufman@gmail.com (E.L.K.); ashleeadams101@gmail.com (A.M.A.); 2 Livestock Issues Research Unit, USDA-ARS, Lubbock, TX 79403, USA; jeff.carroll@ars.usda.gov (J.A.C.); rand.broadway@ars.usda.gov (P.R.B.); 3 Department of Animal and Food Sciences, Texas Tech University, Lubbock, TX 79415, USA; michael.ballou@ttu.edu

**Keywords:** antibody, beef calves, physiological stress, vaccination

## Abstract

**Simple Summary:**

Determining effects of physiological stress and vaccination type on performance and immune responses in cattle can be a difficult task due to the many factors that contribute to the stress response. Nonetheless, defining alterations caused by the stress response after vaccination upon feedlot arrival is vital in improving vaccination practices in the feedlot industry. Utilizing a stress model with stressors induced by current industry practices to mimic a “high-risk” cattle situation, we found that vaccination with a killed or modified-live virus vaccine induces altered immune responses in cattle that underwent industry stressors when compared to a control group. In addition, performance variables were altered in stressed cattle and those that received modified-live vaccination. We propose the feedlot industry consider these results when implementing a vaccine protocol in “high risk”, or chronically stressed, cattle during arrival processing.

**Abstract:**

The study objective was to determine if a combined weaning and transportation stress model affected performance, antibody, endocrine, or hematological responses to modified-live virus (MLV) or killed virus (KV) respiratory vaccination in beef steers. In total, 48 calves (Day 0 BW = 226 ± 6.2 kg) from a single origin were used in a 2 × 2 factorial to evaluate main effects of stress model, vaccine type, and their interaction, resulting in four treatments (*n* = 12/treatment) including non-stress control (C) with KV (CKV), C with MLV (CMLV), stress model implementation (S) with KV (SKV), and S with MLV (SMLV). The C calves were weaned at the origin ranch on Day −37 and transported 472 km to the study site on Day −21 to allow acclimation. The S calves were weaned on Day −3, transported 460 km to a research facility on Day −2, held overnight, and transported 164 km to the study site on Day −1 to mimic the beef cattle marketing process. Vaccines were administered on Day 0 and KV was revaccinated on Day 14. The animal was the experimental unit and dependent variables were analyzed using PROC MIXED with repeated measures (day). A stress model effect (*p* = 0.01) existed for DMI from Day 0 to Day 7 with greater DMI for C (6.19 vs. 4.64 kg/day) when compared to S. The MLV groups had reduced (*p* = 0.05) ADG from Day 0 to Day 56, compared to KV. There was a vaccine type × day (*p* < 0.01) interaction with increased (*p* ≤ 0.01) PI3V- and IBRV-specific antibody titers for KV on Day 21; conversely, MLV had increased (*p* ≤ 0.01) BVDV titers on Days 14, 28, 35, 42, 49, and 56. Increased (*p* ≤ 0.05) BRSV titers were observed in a stress model × day (*p* < 0.01) interaction for S on Days 21, 28, 36, and 42; however, C exceeded S in BVDV-specific antibody concentration on Days 21, 28, and 49. A day effect (*p* < 0.01) was observed for serum haptoglobin with the greatest (*p* < 0.01) concentration on Day 3. Serum cortisol concentration was greater (*p* ≤ 0.04) for C vs. S on Days −2, 0, 1, 3, and 5. Total leukocytes were decreased for C vs. S on Days 0, 1, 3, 5, 7, 14, and 21 (*p* ≤ 0.02). A reduction (*p* ≤ 0.04) in total leukocytes was observed for MLV on Days 5, 7, and 14 vs. KV. Neutrophils and neutrophil:lymphocyte were markedly increased (*p* ≤ 0.01) for S on Day −2, whereas neutrophils were decreased (*p* ≤ 0.01) on Days 1 and 21 for S. Monocytes were decreased on Days 1, 5 and 7 for MLV (*p* ≤ 0.04) and Days −2 to 14 for S (*p* ≤ 0.03). Eosinophils were reduced (*p* = 0.007) for S vs. C on Day −2, yet a distinct rebound response (*p* = 0.03) was noted for S on Day 0. The results indicate that S and MLV vaccination more profoundly induced immunomodulation in beef calves.

## 1. Introduction

Cattle may be vaccinated with an array of antigens and two primary antigen types (i.e., killed or modified-live versions) with the goal of preventing infectious diseases; however, host-dependent vaccine failure may be attributable to parasitic infection, poor health or nutrition status, and/or physiological stress [1]. Acute stress is of shorter duration and has been proposed to stimulate the immune system and increase resistance against infection [2,3]. Chronic stress manifests when an animal experiences a prolonged insult to its homeostatic state with potential to shift the stress response from one that is preparatory to one that is immunosuppressive [3,4,5]. Immunosuppression initiated by sustained glucocorticoid synthesis has been reported to result in transient neutrophilia due to diminished L-selectin-mediated neutrophil migration [6,7], lymphopenia from inhibition of mitosis and redistribution to lymphoid tissue [5,8], eosinopenia due to cellular apoptosis [9], and anti-inflammatory effects by inhibiting synthesis and release of pro-inflammatory cytokines [10]. Therefore, there may be negative consequences when vaccinating chronically stressed cattle, yet the effects may differ for killed virus (KV) vs. modified-live virus (MLV) versions [11]. The current study objective was to determine the effect of physiological stress model on immunological, endocrine, and acute phase protein (APP) responses in beef calves administered pentavalent respiratory vaccines containing replicating MLV or non-replicating KV. Our hypothesis was that immune, endocrine, APP, and performance responses are altered by stress and vaccine antigen type.

## 2. Materials and Methods

This study was conducted from November 2016 to March 2017 at 2 research sites. The initial Day −2 collection sample reported herein for the stress model was conducted at the USDA-ARS Livestock Issues Research Unit near Lubbock, TX, USA. Subsequent samples were collected at the West Texas A&M University (WTAMU) Research Feedlot in Canyon, TX, USA. Animal procedures and experimental protocols were independently approved by the animal care and use committees at USDA-ARS (protocol # 1603S) and WTAMU (protocol # 2-11-16), specific to procedures conducted at each location.

### 2.1. Animals and Housing

Forty-eight clinically heathy, previously unvaccinated Angus × Hereford beef steers (Day 0 average BW = 226 ± 6.2 kg) that were seronegative on Day −37 to infectious bovine rhinotracheitis (IBRV), bovine viral diarrhea virus (BVDV), parainfluenza-3 virus (PI3V), and bovine respiratory syncytial virus (BRSV) antibodies were acquired for use in the study from a single ranch located in central New Mexico. In addition, on Day −37, non-stress control (C) steers were weaned and placed in an isolated pen at the origin ranch where they were allowed ad libitum access to hay and water until Day −21 when they were transported in a sanitized trailer to the WTAMU Research Feedlot (Canyon, TX, USA). The C steers were classified as such because they were weaned, relocated, and acclimated prior to vaccination with handling for sample collection being the primary stressor imposed. Upon arrival to WTAMU, C steers were allotted to their assigned pens where they resided for the remainder of the study. Steers allocated to the stress model treatment (S) were not separated from their dams until Day −3 and were transported on Day −2 approximately 450 km in a sanitized trailer to the USDA-ARS Bovine Immunology Research and Development (BIRD) facility (Lubbock, TX, USA). Upon arrival to BIRD, S calves were allowed to rest overnight in covered soil-surfaced pens with ad libitum access to hay and water. The calves were transported approximately 177 km on Day −1 in a sanitized trailer to the WTAMU Research Feedlot. Upon arrival to the WTAMU Research Feedlot, the S calves were held in soil-surfaced pens separate from the C calves. Simultaneous weaning, transportation, handling, and relocation imposed on S calves over a period of three days was intended to mimic the stressors encountered during a typical marketing process that occurs for high-risk beef calves prior to feedlot arrival and associated chronic stress [12,13,14].

### 2.2. Feed Delivery and Health Observation

Feed bunks were observed each morning (07:00 h) by trained WTAMU Research Feedlot personnel and daily feed offering was adjusted to promote slick bunk management protocol. At the end of each weekly period, feed refusals were weighed, converted to DM basis using the previous day ration DM determined from drying samples in a forced-air oven at 60 °C for 24 h and weekly DMI was adjusted accordingly on a per pen basis. Calves were fed a common starter diet formulated to meet or exceed nutrient requirements of growing beef cattle [15] upon arrival to WTAMU. Calves were observed daily (07:30 h) for signs of respiratory illness, including depression, nasal discharge, isolation from pen mates, anorexia, and gaunt appearance. Those with observed clinical signs were removed from their pen, restrained in a hydraulic squeeze chute (Daniels Manufacturing Co., Ainsworth, NE, USA) and treated with an antimicrobial if their rectal temperature exhibited fever determined by ≥40 °C. A predetermined antimicrobial treatment regimen was established such that initial treatment included administration of ceftiofur crystalline free acid (Excede, 6.6 mg/kg of BW; Zoetis, Kalamazoo, MI, USA). Treated cattle were re-evaluated 120 h later, but no cattle qualified for a second or third antimicrobial treatment, as indicated by feedlot protocol, in this study. Cattle were immediately returned to their home pen following antimicrobial treatment.

### 2.3. Treatments and Vaccination

On Day 0, steers assigned to C and S were randomly and equally allocated to receive either a pentavalent MLV respiratory vaccine (Pyramid 5; Boehringer Ingelheim Animal Health, Inc. (BIAH), Duluth, GA, USA) or pentavalent KV respiratory vaccine (Triangle 5; BIAH). Both vaccines were administered s.c. at dose of 2 mL in the neck, per label recommendation, at 06:00 h on Day 0. On Day 14, per label directions, the KV treatment was administered a secondary booster with KV vaccine in the same manner as the primary dose. The MLV treatment was not subsequently revaccinated. The handling and administration of vaccines used in this study closely followed Beef Quality Assurance guidelines. This resulted in four experimental treatments arranged as a 2 × 2 factorial design consisting of: (1) non-stress control (C) with KV (CKV); (2) C with MLV (CMLV); (3) stress model implementation (S) with KV (SKV); and (4) S with MLV (SMLV). Both vaccines consisted of IBRV, BVDV Type I and II, PI3V, and BRSV antigens, but with replicating (MLV) or non-replicating (KV) properties. Treatment randomization was achieved by drawing labeled cards from a hat with treatment designation of KV or MLV resulting in 12 animals assigned to each of the four treatments.

After vaccine treatments were administered, steers were allotted to one of eight pens. Each treatment was housed in three consecutive pens, resulting in three pens per treatment with four animals per pen. Between each third pen (i.e., between treatments), a pen was left empty to keep treatment groups separated. This biosecurity consideration was implemented to reduce the risk of transmission of viruses from animals that might have shed vaccine-origin virus between stress model or vaccine type.

### 2.4. Blood Collection and Serology

A 4-mL anti-coagulated blood sample was collected on Day −2 at the WTAMU Research Feedlot for C calves and at the USDA-ARS BIRD facility for S calves via jugular venipuncture into tubes containing 7.2 mg EDTA (Vacutainer SST; Becton, Dickinson and Company, Franklin Lakes, NJ, USA). The timing of Day −2 blood collection began at 18:00 h and was coordinated between the two research sites. Subsequent anti-coagulated blood samples were collected at the WTAMU Research Feedlot on Days 0, 1, 3, 5, 7, 14, and 21 and analyzed using an automated hematology analyzer (Idexx, ProCyte Dx Hematology Analyzer, Westbrook, ME, USA) at the WTAMU Animal Health Laboratory to determine complete blood count.

Jugular blood was also collected into 9-mL evacuated blood tubes with no additive (Vacutainer SST; Becton, Dickinson and Company, Franklin Lakes, NJ, USA) to harvest serum used to determine haptoglobin and cortisol concentration. All blood samples were placed in an insulated cooler immediately after sample collection without ice to achieve storage temperature of approximately 20 °C during cold ambient temperature and immediately transported to the WTAMU Animal Health Laboratory. The samples were allowed to clot ≥30 min before centrifugation at 1250× *g* for 20 min at 20 °C. After centrifugation, serum was harvested and stored in triplicate aliquots at −20 °C until subsequent analyses were performed.

One aliquot of the frozen sera was packaged on ice and transported to the Texas A&M Veterinary Medical Diagnostic Laboratory (TVMDL) located in Amarillo, TX, US to determine IBRV-, BVDV-, PI3V-, and BRSV-specific antibody titers from Days 0, 7, 14, 21, 28, 35, 42, 49, and 56. Antibody titers were determined using the virus neutralization assay previously described by [16]. Sera from Days −2, 0, 1, 3, 5, 7, and 14 were used to determine haptoglobin (Hp) concentration at the WTAMU Animal Health Laboratory via a commercial, bovine-specific ELISA kit (Immunology Consultants Laboratory, Inc., Portland, OR, USA) with intra- and inter-assay CV of 8.46% and 11.53%, respectively. Cortisol concentration was determined from sera on Days −2, 0, 1, 3, 5, and 7 at the WTAMU Animal Health Laboratory via commercial EIA kit (Arbor Assays, LLC, Ann Arbor, MI, USA) with intra- and inter-assay CV of 8.62% and 15.50%, respectively.

### 2.5. Statistical Analyses

This 2 × 2 factorial experiment used animal within pen as the experimental unit for average daily gain, body weight, virus-specific antibody response, serum haptoglobin and cortisol, and complete blood count analyses, and pen as the experimental unit for DMI calculations in a completely randomized design [17]. Growth data (ADG, BW, and DMI) were analyzed using the MIXED procedure of SAS (SAS Inst. Inc., Cary, NC, USA). The model for these variables included main effects of stress model, vaccine type and the two-way (stress model × vaccine type) interaction. Data derived from serum samples (antibody titer, Hp, and cortisol) and hematological variables were analyzed using the MIXED procedure of SAS with repeated measures. The model for these repeated variables included main effects of stress model, vaccine type, day, and the two-way (stress model × day; vaccine type × day) and three-way (stress model × vaccine type × day) interactions. The repeated statement was day, and the covariance structure with the lowest Akaike information criterion for each dependent variable was used. Antibody titers were log₂-transformed prior to statistical analysis with consequent results shown. Serum Hp, cortisol, and CBC were tested for normal distribution using the UNIVARIATE procedure and the Shapiro–Wilk test within it to determine if dependent variables were normally distributed. Nonparametric data were log₂-transformed and again tested for normal distribution; if log₂ transformation resulted in a normal distribution, the log-transformed data were statistically analyzed, and back-transformed means were subsequently generated and shown. If log₂ transformation did not result in a normal distribution, non-transformed data were analyzed. Statistical significance was established for main effects of stress model, vaccine type, day, and each possible interaction if the resulting *p*-value was ≤0.05 and statistical tendency was considered if the resulting *p*-value was ≤0.10 and ≥0.06. Differences of least squares means were determined using the PDIFF option in SAS with α = 0.05.

## 3. Results and Discussion

### 3.1. Health and Performance

Only one steer (SMLV treatment) was identified as clinically ill and treated once with an antimicrobial during the study period and this animal died on Day 10; the cause of death was attributed to peritonitis and not determined to be influenced by the experimental treatment. Dry matter intake and ADG are represented in Table 1 and interim and overall BW is represented in Table 2. No BW differences were observed between the C and S groups upon arrival to WTAMU. We did observe a stress model effect (*p* < 0.01) for DMI, from Day 0 to Day 7 such that C calves had greater (*p* < 0.01) DMI than S calves. This was probably a consequence of the acclimation period and bunk training C calves were afforded beginning on Day −21, but also differences in stressors imposed upon initiation of the study. A main effect of stress (*p* < 0.02) on DMI was detected from Day 0 to Day 14 with C exceeding S calves and a tendency (*p* < 0.08) for C to have greater DMI was also noted from Day 0 to Day 28. A stress model × vaccine type interaction (*p* < 0.04) existed for ADG from Day 21 to 28 and a tendency (*p* < 0.10) from Day 0 to Day 28 with CKV having greater (*p* ≤ 0.05) ADG than CMLV. Additionally, vaccine type affected ADG from Day 28 to Day 35 (*p* < 0.04), from Day 35 to Day 42 (*p* < 0.01), from Day 42 to Day 49 (*p* < 0.02), and from Day 0 to Day 56 (*p* < 0.05), illustrating consistent increase in ADG for calves administered KV compared to those administered MLV.

### 3.2. Virus-Specific Antibody Response

**Figure 1:**
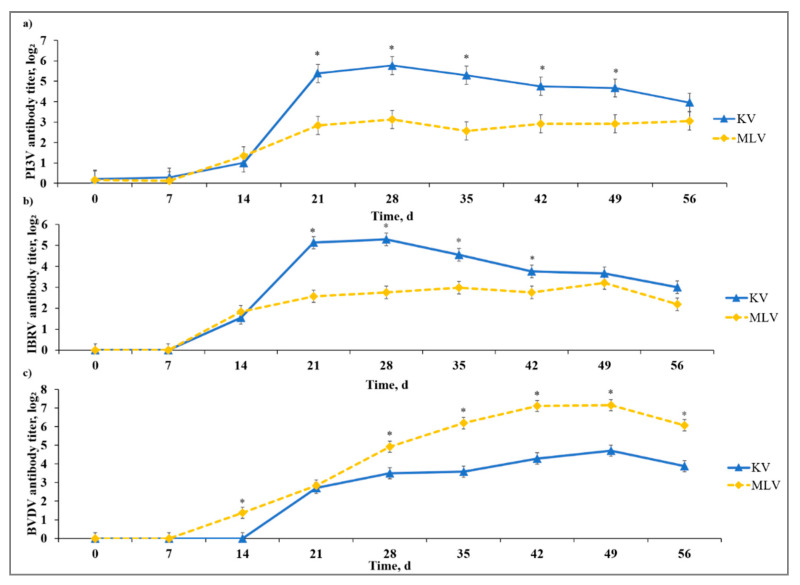
Effect of killed virus (KV; Triangle 5; Boehringer Ingelheim Animal Health, Inc. (BIAH)) and modified-live virus vaccination (MLV; Pyramid 5; BIAH) on serum antibody concentration against: (**a**) parainfluenza-3 virus (PI3V); (**b**) infectious bovine rhinotracheitits virus (IBRV); and (**c**) bovine viral diarrhea virus (BVDV) after subcutaneous injection of multivalent respiratory vaccine on Day 0 and KV booster on Day 14. Effect of vaccination on serum PI3V-specific antibody titer concentration (vaccine treatment × day effect; *p* < 0.01); serum IBRV-specific antibody titer concentration (vaccine treatment × day effect; *p* < 0.01); serum BVDV-specific antibody titer concentration (vaccine treatment × day effect; *p* < 0.01). * KV differs from MLV within day, *p* ≤ 0.02.

All calves used in this study were seronegative to IBRV, BVDV, PI3V, and BRSV on Day −37. We did not detect a stress model × vaccine type interaction or a stress model × vaccine type × day interaction for any of the virus-specific antibody titers evaluated in the current study. There was a vaccine type × day interaction (*p* < 0.01) for PI3V-specific neutralizing antibodies detected in serum such that KV calves had greater (*p* ≤ 0.01) PI3V antibody titer than MLV from Day 21 to Day 49 (Figure 1). Likewise, a vaccine type × day interaction (*p* < 0.01) existed for IBRV-specific neutralizing antibodies such that KV calves exhibited greater (*p* ≤ 0.02) IBRV antibody titer than MLV from Day 21 to Day 42.

There was a vaccine type × day interaction (*p* < 0.01) observed for BVDV-specific antibody (Type Ia; Singer strain); in contrast with PI3V and IBRV observations, the MLV calves had increased (*p* ≤ 0.01) BVDV antibody titer on Days 14, 28, 35, 42, 49, and 56. Temporal trends for all vaccine antigens were indicative of a typical vaccine response with antibody becoming detectable between Days 7 and 14, and peak antibody titers observed between Days 28 and 42 after initial vaccine administration. The authors of [18] reported cattle that received at least one administration of a MLV vaccine had greater virus-neutralizing antibody titers against BVDV than cattle that only received a KV vaccine. This corresponds with our results as MLV calves overall yielded greater BVDV-specific antibody titers throughout the sampling period. In addition, the authors of [19] tested several commercially available MLV and KV vaccines and, while BVDV-specific antibodies from KV peaked on Day 28 and persisted until 84 days post-vaccination, MLV had BVDV-specific antibodies remaining up to 140 days post-vaccination.

**Figure 2:**
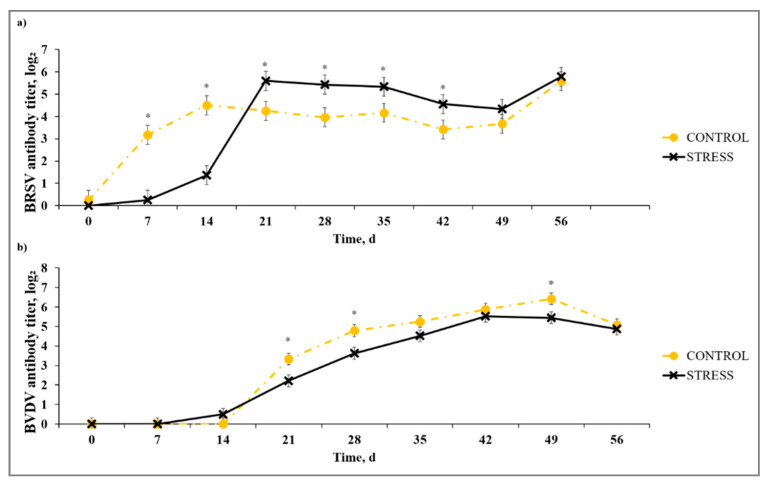
Effect of non-stress control (C; weaned on Day −37; transported to West Texas A&M University (WTAMU) research facility on Day −21; vaccination on Day 0) and stress model implementation (S: weaned on Day −3; transported to USDA-ARS Livestock Issues Research Unit on Day −2; relocated to WTAMU on Day −1; vaccination on Day 0) on serum antibody concentration against: (**a**) bovine respiratory syncytial virus (BRSV); and (**b**) bovine viral diarrhea virus (BVDV). Effect of non-stress control and stress model implementation on serum BRSV-specific antibody titer concentration (stress model × day effect; *p* < 0.01); serum BVDV-specific antibody titer concentration (stress model × day tendency; *p* < 0.09). * C differs from S within day, *p* ≤ 0.02.

A stress model × day interaction (*p* < 0.01) was noted for serum BRSV-specific antibody titer (Figure 2). While C had greater (*p* ≤ 0.01) BRSV titer than S on Days 7 and 14, S exceeded (*p* ≤ 0.05) the C group for BRSV titer on Days 21, 28, 36, and 42 after primary vaccination. This subsequent increase in BRSV titer for the S model may be indicative of greater antibody response in S calves that are experiencing greater immunosuppression. The authors of [11] reported an increase in IBRV- and BVDV-specific antibody response to MLV vaccine agents in animals that concurrently received a chronic stress challenge using repeated dexamethasone injection. A stress model × day interaction (*p* < 0.01) existed for BVDV-specific antibody titer. In contrast to BRSV antibody results, BVDV antibody titer for C exceeded (*p* ≤ 0.03) S on Days 21, 28, and 49. These results contradict results reported in [20], where the BVDV antibody response was enhanced in vaccinated cattle concurrently administered adrenocorticotropic hormone, and those in [11], where the BVDV-specific antibody titer was increased for calves administered acute or chronic dexamethasone treatment vs. control that did not receive dexamethasone concurrent with MLV vaccination. These differences may be due to differences in natural vs. artificial stress model implementation; however, the contrasting results necessitate further exploration of stress and effects on host-viral replication after MLV or KV vaccination.

### 3.3. Serum Haptoglobin

**Figure 3:**
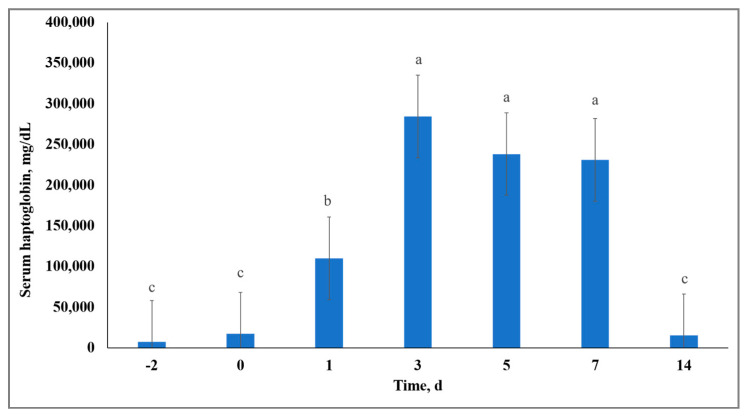
Effect of day on serum haptoglobin (Hp) concentration. Serum Hp concentration (day effect; *p* < 0.01). Across day, unlike superscript letters indicate a difference (*p* ≤ 0.01) in means.

Synthesis of APP primarily by hepatocytes can occur in response to exposure to stressors that result in acute inflammation [21]. Therefore, alterations in APP have been recognized as indicators of cattle morbidity [22], stress [23,24], and inflammation [25]. Although no treatment × day interactions (*p =* 0.68) were observed for serum Hp activity, there was a day effect (*p* < 0.01) resulting in overall serum Hp concentration being increased on Days 3, 5, and 7, and decreased on Days −2, 0, and 14 (Figure 3). This overall Hp response was likely influenced by inflammatory effects of vaccination of the calves on Day 0 [26] and the stress models imposed. However, serum Hp concentration differed from results reported by the authors of [11], who observed acute stress to yield greater synthesis of Hp when compared to chronic stress, whereas we observed no differences in Hp between C and S. These differences may be due to different approaches to stress model implementation; the authors of [11] utilized dexamethasone as a stress challenge that likely induced a robust anti-inflammatory and immunosuppressive effect, whereas we utilized natural stress models in attempt to induce physiological stress.

### 3.4. Serum Cortisol

**Figure 4:**
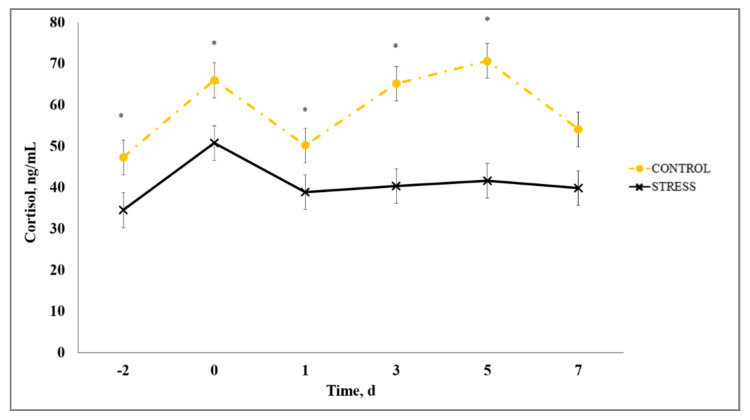
Effect of non-stress control (C; weaned on Day −37; transported to West Texas A&M University research facility on Day −21; vaccination on Day 0) and stress model implementation (S: weaned on Day −3; transported to USDA-ARS Livestock Issues Research Unit on Day −2; relocated to WTAMU on Day −1; vaccination on Day 0) on serum cortisol concentration. Serum cortisol concentration C vs. S (stress model × day interaction; *p* < 0.01). * C differs from S within day, *p* ≤ 0.05.

The primary hormone synthesized and released upon exposure to stressors is cortisol and it has historically been accepted as the gold standard to indicate physiological stress in cattle [27,28] due to its easily accessible peripheral measure that provides a reliable stress response indication [29]. A stress model × day interaction existed (*p* < 0.01) for serum cortisol such that C had increased (*p* ≤ 0.04) serum cortisol concentration on Days −2, 0, 1, 3, and 5 (Figure 4). The timing of the initial stress event and associated cortisol concentration is important to consider. The reduced cortisol observed for S may be influenced by the negative feedback mechanism of the hypothalamus–pituitary–adrenal (HPA) axis such that cortisol concentration may have reached nadir in S calves when they were weaned and transported on Day −3, prior to the initial sample collected on Day −2. Although serum cortisol concentration was not measured on Day −3, our interpretation is further supported by a marked increase in circulating neutrophils on Day −2 for S. Previous research demonstrates that neutrophils in the blood are increased transiently in response to stress and HPA activation due to the negative effect of cortisol on L-selectin expression [6,30]. The authors of [28] reported that, during times of intense or chronic stress, serum cortisol can return to baseline concentration in as little as 90 min after induction of the stressor due to the negative feedback cortisol has on the HPA axis. Negative feedback occurs by inhibiting synthesis and/or secretion of corticotropin-releasing hormone, vasopressin, and adrenocorticotropic hormone, and perhaps decreased mRNA expression of corticotropin-releasing hormone [31]. Our findings in the current study suggest that serum cortisol concentration may not correspondingly indicate the degree of stress in cattle during times of prolonged and/or intensive exposure to stressors.

### 3.5. Complete Blood Count

**Figure 5:**
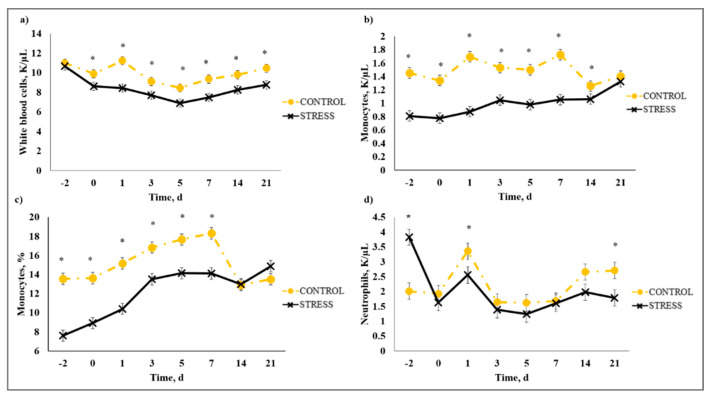
Effect of non-stress control (C; weaned on Day −37; transported to West Texas A&M University research facility on Day −21; vaccination on Day 0) and stress model implementation (S: weaned on Day −3; transported to USDA-ARS Livestock Issues Research Unit on Day −2; relocated to WTAMU on Day −1; vaccination on Day 0) on: (**a**) white blood cells (WBC); (**b**) monocytes (MONO); (**c**) percent monocytes (PERMONO); and (**d**) neutrophils (NEU). Effect of stress model implementation on WBC (stress model × day effect; *p* < 0.01); MONO (stress model × day effect; *p* < 0.01); PERMONO (stress model × day effect; *p* < 0.01); NEU (stress model × day effect; *p* < 0.01). * C differs from S within day, *p* ≤ 0.02.

**Figure 6:**
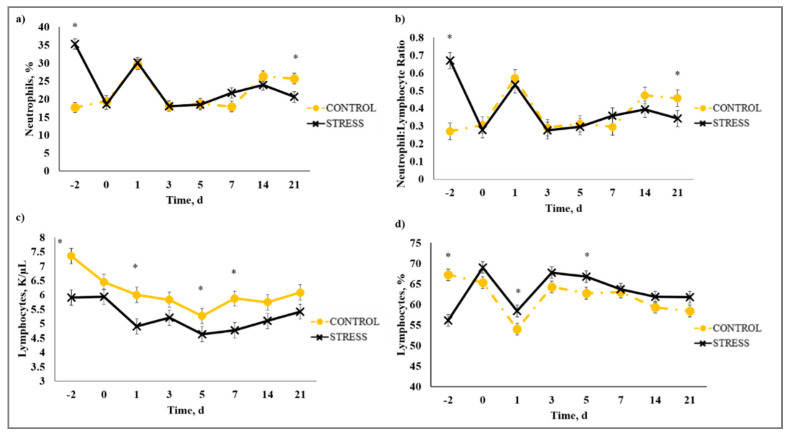
Effect of stress-model control (C; weaned on Day −37; transported to West Texas A&M University research facility on Day −21; vaccination on Day 0) and stress model implementation (S: weaned on Day −3; transported to USDA-ARS Livestock Issues Research Unit on Day −2; relocated to WTAMU on Day −1; vaccination on Day 0) on: (**a**) percent neutrophils (PERNEU); (**b**) neutrophil:lymphocyte ratio (N:L); (**c**) lymphocytes (LYM); and (**d**) percent lymphocytes (PERLYM). Effect of stress model implementation on PERNEU (stress model × day effect; *p* < 0.01); N:L (stress model × day effect; *p* < 0.01); LYM (stress model × day effect; *p* < 0.01); PERLYM (stress model × day effect; *p* < 0.01). * C differs from S within day, *p* ≤ 0.02.

**Figure 7:**
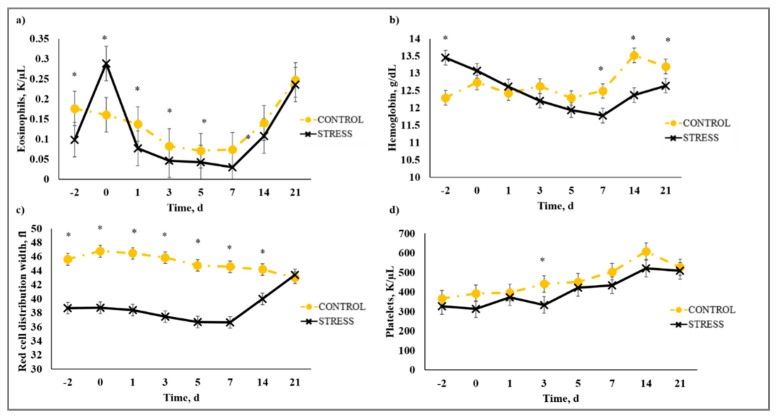
Effect of non-stress control (C; weaned on Day −37; transported to West Texas A&M University research facility on Day −21; vaccination on Day 0) and stress model implementation (S: weaned on Day −3; transported to USDA-ARS Livestock Issues Research Unit on Day −2; relocated to WTAMU on Day −1; vaccination on Day 0) on: (**a**) eosinophils (EOS); (**b**) hemoglobin (HGB); (**c**) red cell distribution width (RDW); and (**d**) platelets (PLT). Effect of stress model implementation on EOS (stress model × day effect; *p* < 0.01); HGB (stress model × day effect; *p* < 0.01); RDW (stress model × day effect; *p* < 0.01); PLT (stress model × day effect; *p* < 0.02). * C differs from S within day, *p* ≤ 0.05.

**Figure 8:**
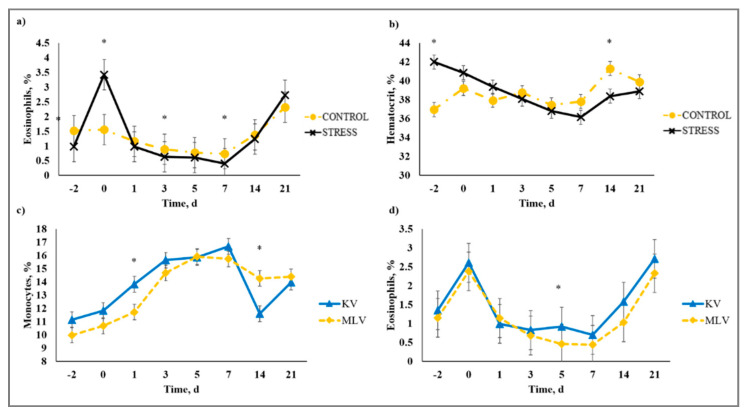
Effect of non-stress control (C; weaned on Day −37; transported to West Texas A&M University research facility on Day −21; vaccination on Day 0) and stress model implementation (S: weaned on Day −3; transported to USDA-ARS Livestock Issues Research Unit on Day −2; relocated to WTAMU on Day −1; vaccination on Day 0) on: (**a**) percent eosinophils (PEREOS); and (**b**) hematocrit (HCT). Effect of stress model on PEREOS (stress model × day effect; *p* < 0.01); HCT (stress model × day effect; *p* < 0.01) and effect of killed virus (KV; Triangle 5; Boehringer Ingelheim Animal Health, Inc. (BIAH)) and modified-live virus vaccination (MLV; Pyramid 5; BIAH) treatment on: (**c**) percent monocytes (PERMONO); and (**d**) percent eosinophils (PEREOS) after subcutaneous injection of multivalent respiratory vaccine on day 0. Effect of vaccination on PERMONO (vaccine treatment × day effect; *p* < 0.01); PEREOS (vaccine treatment × day effect; *p* < 0.01). * C differs from S within day, *p* ≤ 0.02; * KV differs from MLV within day, *p* ≤ 0.02.

There was a stress model × day interaction (*p* < 0.01) observed for total leukocytes (WBC), neutrophils (NEU), lymphocytes (LYM), neutrophil to lymphocyte ratio (N:L), monocytes (MONO), eosinophils (EOS), percent neutrophils (PERNEU), percent lymphocytes (PERLYM), percent monocytes (PERMONO), percent eosinophils (PEREOS), hemoglobin (HGB), hematocrit (HCT), mean corpuscular volume (MCV), mean cell hemoglobin (MCH), mean cell hemoglobin concentration (MCHC), red cell distribution width (RDW), and platelets (PLT). Increased (*P* ≤ 0.02) WBC concentrations were observed for C relative to S steers beginning on Day 0 and continued through Day 21 (Figure 5). A similar profile was observed for MONO and PERMONO such that MONO were increased (*p* ≤ 0.04) for C calves from Day −2 to Day 14 and PERMONO were increased (*p* ≤ 0.01) for C from Day −2 to Day 7. The greatest (*p* < 0.01) total WBC was observed in C steers on Day 1 (11.22 K/µL) followed by a decrease until Day 5 (8.45 K/µL). This corresponds with results reported in [32], where increased WBC from 24 to 48 h post-vaccination was followed by a decrease 72 h post-vaccination in animals that were administered a single dexamethasone injection intended to represent acute stress. While it has been proposed that short-term stress induces an initial increase followed by a decrease in blood leukocyte concentrations [33], the overall increase in WBC for C compared to S calves may be indicative of the greater serum cortisol concentration observed for C calves during the early part of this study. The authors of [34] reported enhanced activation of hematopoietic stem cell production for neutrophils and monocytes in mice with increased corticosterone hormone levels. The initial increase followed by a decrease in WBC for C calves was primarily influenced by the concentration of NEU and LYM. An increase in circulating NEU was observed such that C calves exhibited greater (*p* < 0.01) NEU on Day 1 (3.36 K/µL) compared to S (2.00 K/µL) followed by a decrease in NEU for C on Day 3 (1.64 K/µL). However, S calves had previously exhibited a marked increase (*p* < 0.01) in NEU on Day −2 (3.83 K/µL) followed by a decrease on Day 0 (1.63 K/µL). This response is indicative of a rebound effect on Day 0 for S following the marked increase in NEU on Day −2. Similar results were observed for PERNEU and N:L with the greatest (*p* < 0.01) N:L for S steers on Day −2 (Figure 6). There was an additional PERNEU increase (*p* < 0.01) on Day 1 for both C and S steers. No further differences were reported for PERNEU or N:L until Day 21 when increased (*p* ≤ 0.03) PERNEU and N:L were observed for C calves. Decreased (*p* ≤ 0.04) LYM were observed on Days -2, 1, 5, and 7 for S calves, whereas PERLYM was decreased (*p* < 0.01) on Day −2 for S calves with increased PERLYM on Days 1 (*p* < 0.04) and 5 (*p* < 0.05). A marked increase (*p* ≤ 0.02) was observed for EOS (Figure 7) and PEREOS (Figure 8) on Day 0 in S after they were observed to be less (*p* ≤ 0.01) on Day −2. Subsequently, decreased (*p* ≤ 0.03) EOS in S was observed on Day 1, 3, 5, and 7 and decreased (*p* ≤ 0.01) PEREOS existed on Day 3 and 7. Additionally, there was >50% decrease (*p* ≤ 0.01) in EOS and PEREOS from Day 0 to Day 7 for both C and S calves. The eosinopenia after stress model implementation corresponds with results in water restricted and transported steers [35] and may also be influenced by viral vaccination and glucocorticoid release from stress and handling as glucocorticoids are known to decrease EOS concentrations [32]. Dexamethasone administration has been reported to decrease LYM [6,32,36] and EOS [5,32,36] concentrations in the blood of cattle. This agrees with our results, as S calves exhibited decreased (*p* ≤ 0.04) LYM and EOS compared to C calves which may be the result of greater redistribution of LYM to lymphatic tissue, apoptosis and decreased proliferation of LYM [36], and a speculated decrease in metabolic function for EOS [5] from intense endogenous glucocorticoid release. However, it is important to note that S calves did not exhibit classically defined lymphopenia or eosinopenia as the decreased LYM and EOS concentrations resided in the normal reference range for cattle [37]. Increased (*p* ≤ 0.01) HGB and HCT concentrations were observed on Day −2 for S. In addition, S calves exhibited decreased (*p* ≤ 0.05) HGB on Days 7, 14, and 21 and HCT (*p* < 0.01) on Day 14. Profiles for MCV and MCH were comparable such that S calves consistently exhibited numerically less of each variable than C calves. Decreased (*p* ≤ 0.03) concentrations were observed for MCHC in S calves on Days -2 and 1 and PLT were decreased (*p* < 0.02) for S calves on Day 3. Stress model influenced RDW with decreased (*p* ≤ 0.01) RDW for S calves from Day −2 to Day 14 of the sampling period with similar (*p* > 0.72) values on Day 21. These RBC component responses were most likely indicative of dehydration and disrupted feed intake experienced by S calves from weaning on Day −3 and transportation on Days -2 and -1. Similar results have been reported such that HCT and HGB [35,38] were increased with increased time of feed and water deprivation. Although plasma volume reduction was reported in [38], MCV and MCHC were not greatly affected by feed and water deprivation, thus agreeing with our results.

**Figure 9:**
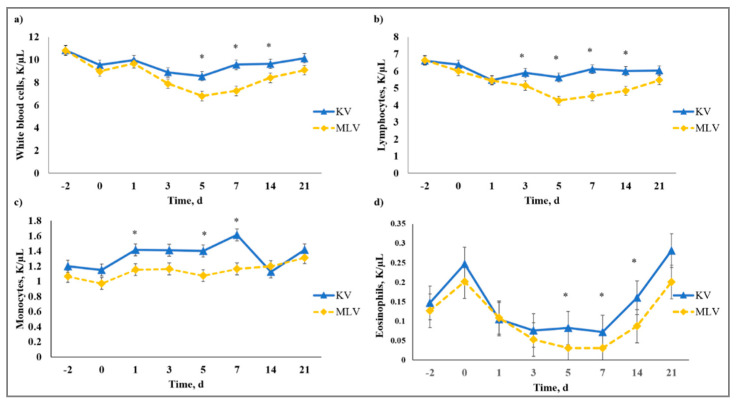
Effect of killed virus (KV; Triangle 5; Boehringer Ingelheim Animal Health, Inc. (BIAH)) and modified-live virus vaccination (MLV; Pyramid 5; BIAH) on: (**a**) white blood cells (WBC); (**b**) lymphocytes (LYM); (**c**) monocytes (MONO); and (**d**) eosinophils (EOS) after subcutaneous injection of multivalent respiratory vaccine on Day 0. Effect of vaccination on WBC (vaccine treatment × day effect; *p* < 0.01); LYM (vaccine treatment × day effect; *p* < 0.01); MONO (vaccine treatment × day effect; *p* < 0.01); EOS (vaccine treatment × day effect; *p* < 0.01). * KV differs from MLV within day, *p* ≤ 0.04.

There was a vaccine type × day interaction (*p* < 0.01) on WBC, LYM, MONO, PERMONO, EOS, PEREOS, HGB, HCT, MCV, MCH, RDW, and PLT. Decreased (*p* ≤ 0.01) WBC were observed from Day 5 to Day 14 for MLV that was influenced by the decreased (*p* ≤ 0.04) LYM observed from Day 3 to Day 14 for MLV calves (Figure 9). Viral replication is generally known to decrease circulating LYM concentration and this phenomenon was observed by [39] in cattle that were challenged with BVDV; however, lymphopenia is most likely due to redistribution of LYM to lymphatic tissue in response to BVDV infection. The MLV calves had decreased (*p* ≤ 0.05) MONO and PERMONO on Day 1, and MONO was decreased (*p* ≤ 0.02) on Days 5 and 7, whereas, MLV had greater (*p* < 0.01) PERMONO than KV calves on Day 14. Monocytopenia observed for MLV could be from viral replication that existed for MLV, but not KV calves until PERMONO was similar (*p* > 0.80) on Day 21 for KV and MLV. Responses detected for EOS and PEREOS were similar such that MLV had reduced (*p* ≤ 0.02) EOS and PEREOS concentrations on Day 5. The decreases in EOS and PEREOS in MLV calves may indicate EOS recruitment to infected sites from viral replication initiated by the replicating antigens in the MLV vaccine. The authors of [40] reported increased EOS recruitment to respiratory syncytial virus (RSV) infected lungs in mice and guinea pigs, suggesting decreased circulating EOS concentrations. Whereas results from MCV, MCH, RDW, and PLT revealed a vaccine type × day interaction, means within day were not different for any of these variables (data not shown).

**Figure 10:**
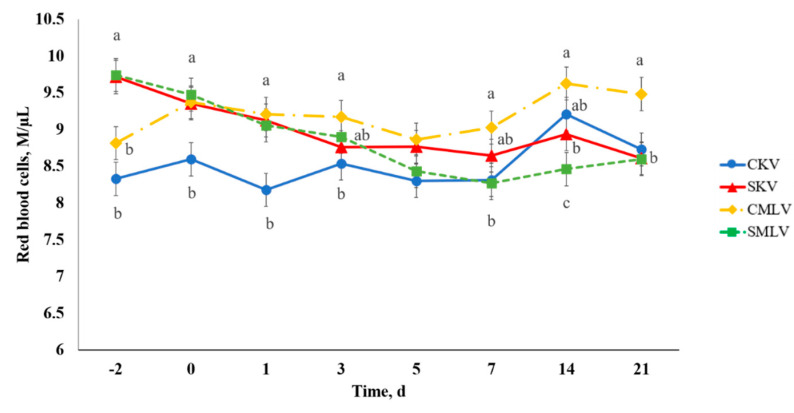
Effect of stress model and vaccination on red blood cells (RBC) after non-stress control (C; weaned on Day −37; transported to West Texas A&M University research facility on Day −21; vaccination on Day 0) and stress model implementation (S: weaned on Day −3; transported to USDA-ARS Livestock Issues Research Unit on Day −2; relocated to WTAMU on Day −1; vaccination on Day 0) implementation and killed virus (KV; Triangle 5; Boehringer Ingelheim Animal Health, Inc. (BIAH)) and modified-live virus vaccination (MLV; Pyramid 5; BIAH) on Day 0. Red blood cells (stress model × vaccine treatment × day effect; *p* < 0.04). Across day, uncommon superscript letters between means indicate a difference (*p* ≤ 0.05).

A stress model × vaccine type × day interaction existed (*p* < 0.04) for RBC (Figure 10). A difference was observed between CKV and CMLV calves such that CMLV had greater (*p* ≤ 0.05) RBC on Days 0–7 and 21. Additionally, SMLV and SKV calves had increased (*p* ≤ 0.05) RBC compared to CKV calves on Days −2, 0, and 1. These results could indicate more dehydration among treatments with elevated RBC because, as previously mentioned, RBC are known to increase during water deprivation in cattle [35,38]. In addition, decreased WBC due to leukocyte migration would additively cause an increase in RBC. The increases in RBC for SMLV and SKV were likely a result of our stress model implementation with disrupted water intake immediately prior to vaccination because calves were transported from the origin ranch to USDA-ARS on Day −2 and to WTAMU on Day −1.

## 4. Conclusions

Our data reveal some alterations in antibody response, APP, endocrine, and hematological variables from implementation of stress model and vaccination with KV or MLV respiratory vaccine. Antigen-specific antibody titer results suggest that KV had a greater antibody response against PI3V and IBRV antigens, whereas MLV had a greater antibody response against BVDV. Stress model implementation yielded inconsistent results to titer concentrations such that BRSV-specific antibody response was delayed in S and BVDV-specific antibody response was greater in C. The stress model may have caused greater stress-induced immunosuppression because increased NEU were observed following stress model implementation and LYM and EOS were reduced, despite overall greater cortisol detected for C. Dry matter intake and ADG was reduced for S and MLV groups and this was probably influenced by differences in inflammation and the associated effect on appetite and growth. Further research investigating the interaction between stress-induced immunosuppression and concurrent use of different vaccine types is needed to better define the safe and efficient use of respiratory vaccines in cattle.

## Figures and Tables

**Table 1:** Effect of stress model and killed or modified-live virus vaccination on feed intake and performance of beef calves.

Item	Treatment ^1^				SEM ^2^	*p*-Value		
	CKV	CMLV	SKV	SMLV		Stress Model	Vaccine Type	Interaction
DMI, kg/day								
Days 0–7	6.20	6.18	4.70	4.57	0.15	0.01	0.61	0.70
Days 7–14	6.16	5.00	5.24	4.89	0.54	0.37	0.20	0.47
Days 14–21	6.99	6.22	6.00	5.98	0.47	0.23	0.43	0.45
Days 21–28	7.80	7.13	7.02	7.06	0.41	0.33	0.47	0.42
Days 28–35	8.00	7.86	7.68	7.83	0.41	0.68	0.98	0.73
Days 35–24	8.46	7.76	7.98	7.49	0.54	0.50	0.30	0.85
Days 42–49	8.66	7.76	8.16	7.76	0.59	0.68	0.30	0.68
Days 49–56	9.38	8.42	8.54	7.97	0.46	0.20	0.13	0.68
Days 0–14	6.18	5.59	4.97	4.73	0.33	0.01	0.24	0.61
Days 0–28	6.79	6.13	5.74	5.62	0.38	0.08	0.34	0.50
Days 0–56	7.71	7.04	6.92	6.69	0.40	0.20	0.30	0.60
ADG, kg/day								
Days 0–7	0.31	0.27	0.31	0.78	0.36	0.46	0.55	0.47
Days 7–14	0.95	0.02	0.98	0.22	2.53	0.96	0.74	0.97
Days 14–21	1.75	2.09	1.08	1.24	2.63	0.76	0.92	0.97
Days 21–28	2.05 ᵃ	1.61 ᵇ	1.85 ᵃᵇ	1.97 ᵃᵇ	0.13	0.54	0.21	0.04
Days 28–35	1.40	1.81	1.28	1.86	0.24	0.88	0.04	0.72
Days 35–24	1.53	0.89	1.34	0.55	0.22	0.23	0.01	0.24
Days 42–49	1.97	1.27	1.83	1.60	0.20	0.62	0.02	0.21
Days 49–56	1.94	1.94	2.02	1.75	0.25	0.81	0.57	0.56
Days 0–14	0.63	−0.15	0.65	0.53	1.29	0.78	0.72	0.79
Days 0–28	1.26	0.85	1.06	1.08	0.14	0.92	0.15	0.10
Days 0–56	1.48	1.16	1.34	1.28	0.10	0.86	0.05	0.18

^1^ CKV = non-stressed control (C; weaned on Day −37; transported to West Texas A&M University Research Feedlot on Day −21; vaccination on Day 0) implementation and killed virus (KV; Triangle 5; Boehringer Ingelheim Animal Health, Inc. (BIAH)); CMLV = C and modified-live virus vaccination (MLV; Pyramid 5; BIAH) on Day 0; SKV = stress model (S: weaned on Day −3; transported to USDA-ARS Livestock Issues Research Unit on Day −2; relocated to WTAMU on Day −1; vaccination on Day 0) implementation and KV; SMLV = S and MLV. Across rows, unlike superscript letters indicate a statistical difference (*p* ≤ 0.05). ^2^ Pooled standard error of the means.

**Table 2:** Effect of stress model and killed or modified-live virus vaccination on body weight of beef calves.

Item	Treatment ^1^				SEM ^2^	*p*-Value		
	CKV	CMLV	SKV	SMLV		Stress Model	Vaccine Type	Interaction
BW, kg								
Day 0	217	239	224	220	11.30	0.60	0.41	0.26
Day 7	219	239	226	226	11.52	0.79	0.38	0.37
Day 14	226	237	233	226	11.75	0.86	0.85	0.42
Day 21	238	252	241	236	11.76	0.57	0.69	0.42
Day 28	252	263	254	250	12.05	0.60	0.77	0.53
Day 35	262	276	263	263	11.97	0.59	0.55	0.57
Day 42	272	282	272	267	12.50	0.52	0.86	0.54
Day 49	286	291	285	273	13.24	0.44	0.78	0.52
Day 56	300	305	299	291	12.98	0.56	0.90	0.62

^1^ CKV = non-stressed control (C; weaned on Day −37; transported to West Texas A&M University Research Feedlot on Day −21; vaccination on Day 0) implementation and killed virus (KV; Triangle 5; Boehringer Ingelheim Animal Health, Inc. (BIAH)); CMLV = C and modified-live virus vaccination (MLV; Pyramid 5; BIAH) on Day 0; SKV = stress model (S: weaned on Day −3; transported to USDA-ARS Livestock Issues Research Unit on Day −2; relocated to WTAMU on Day −1; vaccination on Day 0) implementation and KV; SMLV = S and MLV. ^2^ Pooled standard error of the means.
